# Functional Characterization of Odorant Binding Protein 27 (RproOBP27) From *Rhodnius prolixus* Antennae

**DOI:** 10.3389/fphys.2018.01175

**Published:** 2018-08-23

**Authors:** Daniele S. Oliveira, Nathália F. Brito, Thiago A. Franco, Monica F. Moreira, Walter S. Leal, Ana C. A. Melo

**Affiliations:** ^1^Laboratório de Bioquímica e Biologia Molecular de Vetores, Instituto de Química, Universidade Federal do Rio de Janeiro, Rio de Janeiro, Brazil; ^2^Instituto Nacional de Ciência e Tecnologia em Entomologia Molecular-CNPq, Rio de Janeiro, Brazil; ^3^Department of Molecular and Cellular Biology, University of California, Davis, Davis, CA, United States

**Keywords:** odorant binding proteins, *Rhodnius prolixus*, RNAi, sexual behavior, olfaction

## Abstract

Olfactory proteins mediate a wide range of essential behaviors for insect survival. Odorant binding proteins (OBPs) are small soluble olfactory proteins involved in the transport of odor molecules (=odorants) through the sensillum lymph to odorant receptors, which are housed on the dendritic membrane of olfactory sensory neurons also known as olfactory receptor neurons. Thus, a better understanding of the role(s) of OBPs from *Rhodnius prolixus*, one of the main vectors of Chagas disease, may ultimately lead to new strategies for vector management. Here we aimed at functionally characterize OBPs from *R. prolixus*. Genes of interest were selected using conventional bioinformatics approaches and subsequent quantification by qPCR. We screened and estimated expression in different tissues of 17 *OBPs* from *R. prolixus* adults. These analyses showed that 11 *OBPs* were expressed in all tissues, whereas six *OBP* genes were specific to antennae. Two OBP genes, *RproOBP6* and *RproOBP13*, were expressed in both male and female antennae thus suggesting that they might be involved in the recognition of semiochemicals mediating behaviors common to both sexes, such host finding (for a blood meal). Transcripts for *RproOBP17* and *RproOBP21* were enriched in female antennae and possibly involved in the detection of oviposition attractants or other semiochemicals mediating female-specific behaviors. By contrast, *RproOBP26* and *RproOBP27* might be involved in the reception of sex pheromones given that their transcripts were highly expressed in male antennae. To test this hypothesis, we silenced *RproOBP27* using RNAi and examined the sexual behavior of the phenotype. Indeed, adult males treated with *dsOBP27* spent significantly less time close to females as compared to controls. Additionally, docking analysis suggested that RproOBP27 binds to putative sex pheromones. We therefore concluded that RproOBP27 might be a pheromone-binding protein.

## Introduction

Chemical signals are essential to promote specific behaviors in different species (Gaillard et al., 2004). Insects, in particular, depend on the correct identification of volatile compounds (semiochemicals) for survival and reproduction (Cruz-Lopez et al., 2001; Syed and Leal, 2009; Pitts et al., 2014). *Rhodnius prolixus* is one of the main vectors of the protozoan *Trypanosoma cruzi*, the etiological agent of Chagas disease. According to estimates based on 2010 WHO data, 5,742,167 people in 21 Latin American countries are infected (WHO, 2015). New cases due to vector transmission were estimated to 29,925/year (WHO, 2015). Several proteins participate in insect chemosensation, including odorant binding proteins (OBPs), which transports odor molecules through the sensillum lymph to odorant receptors (ORs) (Fan et al., 2011; Leal, 2012; Brito et al., 2016; Pelosi et al., 2018); ORs located in the membrane of olfactory sensorial neurons (Benton, 2006), which recognize volatile odorant molecules (de Bruyne and Baker, 2008); and ionotropic receptors (IRs), which detect diverse chemical ligands from the environment (Benton et al., 2009). OBPs represent the first contact between semiochemicals from the environment and the olfactory sensory system since they are responsible for transporting hydrophobic ligands to their specific ORs (Wojtasek and Leal, 1999; Fan et al., 2011). OBPs are small soluble proteins secreted by accessory cells into the antenna sensillar lymph surrounding the olfactory sensory neurons (Brito et al., 2016; Pelosi et al., 2018). Initially, OBPs were identified and characterized at molecular level in *Drosophila melanogaster* (Brito et al., 2016). After that, other studies reported that OBPs were identified in different insect species, including the disease vectors *Anopheles gambiae* (Vogt, 2002; Mastrobuoni et al., 2013), *Aedes aegypti* (Zhou et al., 2008), *Culex quinquefasciatus* (Pelletier and Leal, 2009), and *Glossina morsitans morsitans* (Liu et al., 2010). In hemipterans, the first characterized OBP was *Lygus* antennal protein (LAP) from the phytophagous insect *Lygus lineolaris* (Dickens et al., 1998). LAP expression was shown to be adult-specific, initiating development in antennae during the transitional period that precedes adult molt (Vogt et al., 1999). Subsequently, it was reported that in the alfalfa plant bug *Adelphocoris lineolatus*, some OBP genes exhibited high differential expression in male and female antennae (Gu et al., 2011a). More recently, the genome of the hematophagous hemipteran *R. prolixus* was released and has been predicted to encode 27 putative *OBP* genes (Mesquita et al., 2015). However, only 17 OBPs were actually identified in the antenna proteome (Oliveira et al., 2017), suggesting that these proteins could be associated with odor detection. Hemipteran insects have many intricate behaviors such as male aggregation (Vitta et al., 2009; Pontes and Lorenzo, 2012), oviposition aggregation (Rolandi and Schilman, 2017), food ingestion (Diaz-Albiter et al., 2016; Franco et al., 2016), and avoidance behavior (Zermoglio et al., 2015). Despite the importance of *R. prolixus* as a vector of Chagas disease, the role(s) of OBPs in odor recognition has not yet been investigated, even though there is strong evidence that these insects use chemical signals to mediate sexual communication. It is already known that males can be oriented toward air currents carrying volatiles produced by female metasternal glands (MGs) (Pontes et al., 2008, 2014). Recently, several studies have used the RNA interference (RNAi) technique to identify OBP functions in insects (Biessmann et al., 2010; Pelletier et al., 2010; Zhang et al., 2016). Moreover, it might be possible to link behavior to OBP(s) by gene silencing (He et al., 2011; Swarup et al., 2011; Deng et al., 2013; Li et al., 2016; Shorter et al., 2016; Lin et al., 2017). In fact, RNAi based studies have shown that OBPs are involved in the detection of oviposition attractants (Biessmann et al., 2010; Pelletier et al., 2010), plant volatiles (He et al., 2011; Li et al., 2016; Zhang et al., 2016), host molecules (Deng et al., 2013), in the survival of insects (He et al., 2011; Lin et al., 2017), and regulates mating behavior (Shorter et al., 2016). Therefore, the purpose of this study was to investigate the role of the 17 OBPs previously identified in antenna proteome (Oliveira et al., 2017), in *R. prolixus* chemical communication. Results revealed that 11 *OBPs* were expressed in all tissues, whereas six *OBPs* were shown to be antennae-specific. *RproOBP6* and *RproOBP13* were expressed in both male and female antennae. *RproOBP17* and *RproOBP21* were enriched in female antennae. In contrast, *RproOBP26* and *RproOBP27* were significantly expressed in male antennae, which suggests these proteins could play a role in male specific behaviors. Interestingly, *RproOBP26* was also reported overexpressed in the insect gut (Ribeiro et al., 2014), suggesting that RproOBP26 might be involve in multiple roles. The potential role of RproOBP27 in the detection of odorants was further investigated by RNAi because this protein is male antennae-specific and thus a putative pheromone-binding protein. Additionally, docking analysis suggested that RproOBP27 favorably binds the most abundant chemicals (putative sex pheromones) identified in female MGs (Pontes et al., 2008), which indicates this OBP could be involved in the detection of female-derived semiochemicals. In a behavioral assay, males injected with *dsOBP27* spent significantly less time close to females when compared to controls, strongly suggesting RproOBP27 plays a role in the reception of female-derived semiochemicals.

## Materials and Methods

### Insect Rearing

*Rhodnius prolixus* were taken from a colony at Insect Biochemistry Laboratory/Federal University of Rio de Janeiro/Brazil. Insects were maintained at 28°C and 80–90% relative humidity under a photoperiod of 12 h of light/12 h dark. Insects used in this work were unmated males fed on rabbit blood at 3-week intervals. Male *R. prolixus* injected with dsRNA were kept on cages maintained under the same conditions. In dsRNA experiments, unfed male nymphs (5*^th^* instar, N5) were injected with 1 μg of dsRNA (*dsOBP27* or *ds*β-*gal*) diluted in 1 μL of RNase-free water into the metathoracic cavity using a 10 μL Hamilton syringe. Nymphs were fed on rabbit blood 7 days after dsRNA treatment.

### Ethics Statement

All animal care and experimental protocols were conducted following the guidelines of the institutional care and use committee (Committee for Evaluation of Animal Use for Research from Federal University of Rio de Janeiro), which are based on the National Institute of Health Guide and Use of Laboratory Animals (ISBNo-309-05377-3). The protocols were approved by the Committee for Evaluation of Animal Use for Research (CAUAP) from the Federal University of Rio de Janeiro, under register number CEAU-UFRJ#1200.001568/2013-87, 155/13. Technicians dedicated to the animal facility at Federal University of Rio de Janeiro carried out all aspects related to rabbit husbandry under strict guidelines to ensure careful and consistent handling of the animals.

### Tissue Isolation, RNA Extraction, and cDNA Synthesis

Antennae, proboscis, legs, and heads (without antennae and proboscis) from 30 blood-fed male and 30 blood-fed female were dissected using forceps. Tissues were transferred to polypropylene tube separately, frozen in liquid nitrogen and triturated with plastic pestle. Total RNA was extracted from different tissues using TRIzol (Invitrogen, Carlsbad, CA, United States) according to the manufacturer’s instructions. RNA concentrations were determined at 260 nm on a UV-1800 spectrophotometer (Shimadzu, Inc., Kyoto, Japan). RNA integrity was evaluated in 1% agarose gel. RNAs were treated with RNase-free DNAse I (Fermentas International, Inc., Burlington, ON, Canada), 1 μg of RNA was used for cDNA synthesis with High-Capacity cDNA Reverse Transcription kit and random primers (Applied Biosystems, Foster City, CA, United States).

### Spatial Transcript Quantification

Gene sequences of 17 *RproOBPs* were downloaded from *R. prolixus* genome database^1^ for primer design using OligoPerfect^TM^ Designer – Thermo Fisher Scientific tool. All primer sequences are listed in **Supplementary Table S1**. PCR studies were performed using GoTaq^®^ Green Master Mix kit (Promega, Madison, WI, United States). *R. prolixus*’ ribosomal gene 18S (*RproR18S*) was used as the reference gene (Majerowicz et al., 2011). PCRs were performed on Veriti^®^ Thermal Cycler-96 well thermocycler (Applied Biosystems, Foster City, CA, United States), consisting of 35 cycles for *RproOBPs* and 25 cycles for *RproR18S* under the following conditions, 94°C for 3 min, followed by denaturation steps at 94°C for 30 s, annealing temperature was set according to each primer pair (**Supplementary Table S1**) for 30 s and the extension step at 72°C for 1 min and 30 s, finally followed by 72°C for 10 min. cDNA from antennae, proboscis, legs, and heads (without antennae and proboscis) obtained from adults were used as templates for PCR. PCR products were analyzed on a 1% agarose gel stained with GelRed^TM^ (Biotium, Hayward, CA, United States) in TAE buffer pH 8 (40 mM Tris-acetate, 1 mM EDTA). Gels were digitalized on DNR MiniBIS Pro Bio-Imaging Systems (BioAmerica Inc., Miami, FL, United States). qPCRs were performed on a StepOne^TM^ Real-Time PCR System (Applied Biosystems) thermocycler using Power SYBR^®^ Green PCR Master Kit (Applied Biosystems). cDNA from adult antennae, proboscis, legs and heads (without antennae and proboscis) were used as templates for qPCRs. Oligonucleotide concentrations consisted of 400 nM for *RproR18S* and 600 nM for *RproOBPs*. Reactions were carried out in three biological replicates and three technical replicates for each sample, in a 48-well optical plate with the following initial cycle, 50°C for 2 min; 95°C for 10 min; followed by denaturation steps at 94°C for 15 s then 60°C for 15 s and extension at 72°C for 1 min for 40 cycles; dissociation curves were obtained under standard conditions of the instrument. *RproR18S* gene was used as reference gene for the normalization of Ct (threshold cycle) values. The relative gene expression of the *RproOBPs* was determined by 2^-ΔΔ*Ct*^ method (Livak and Schmittgen, 2001). Data were presented as mean ± standard error of three independent experiments in biological triplicates.

### dsRNA Synthesis and Gene Silencing Assays

Fragments of PCR product encoding *RproOBP27*, size 146 bp, were amplified by PCR using cDNA from blood-fed male adults antennae produced as described above. The following conditions were used for amplification: one cycle for 3 min at 94°C, following by 35 cycles of 30 s at 94°C for denaturation, 30 s at 59°C for annealing and the extension step at 72°C for 1 min and 30 s, followed by 72°C for 10 min. The primers used for amplification of templates for dsRNA synthesis are listed at **Supplementary Table S1**. These primers contained a T7 polymerase binding sequence required for dsRNA synthesis. These products were used as the template for the transcription reactions using the enzyme T7 RNA polymerase with MEGAscriptRNAi kit (Ambion, Austin, TX, United States), according to manufacturer’s protocol. The β-*galactosidase protein* (β-*gal*) gene from *Culex quinquefasciatus* (Xu et al., 2014) cloned into pGEM-T (Promega) was amplified by PCR using T7 minimal promoter primers under the following conditions: one cycle for 3 min at 94°C, following by 35 cycles of 30 s at 94°C for denaturation, 30 s at 56°C for annealing and the extension step at 72°C for 1 min and 30 s, followed by 72°C for 10 min. The PCR product generated was used as the template for β-gal dsRNA synthesis used as a control in the silencing assay. Following *in vitro* synthesis, all dsRNAs were purified using phenol-chloroform (1:1), quantified using a spectrophotometer at 260 nm and analyzed by 1% agarose gel electrophoresis stained with GelRed^TM^. RNAi experiments were performed as described by Franco et al., 2016. Briefly, 1 μL of dsRNA (1 μg/μL RNase-free water) was injected into the metathoracic cavity of starved N5 males (*N* = 20 for each dsRNA treatment), using a 10 μL Hamilton syringe, after 7 days insects were blood fed and monitored during 21 days until ecdysis. The resulting dsRNA-treated adults were fed on rabbit blood. In bioassays, insects from the different groups were tested individually.

Starved N5 males treated with dsRNA as described above (*N* = 20 for *dsOBP27* and *N* = 20 for *ds*β-*gal*) were kept under controlled temperature and humidity conditions. Mortality was monitored from the 3*^rd^* to the 20*^th^* day after dsRNA injection. The number of survival N5 in this period was registered. The effect of dsRNA injection on blood feeding was performed as described by Franco et al., 2016. The *dsOBP27*- and *ds*β-*gal*-treated N5s were weighed 2 h before and 2 h after feeding. The ingested mass (mg) was calculated by the weight difference after and before feeding.

### Female Recognition Bioassay

The ability of adult males treated with dsRNA to recognize females was accessed using a bioassay adapted from Zermoglio et al. (2015) (**Supplementary Figure S5**). Adult males injected with dsRNA in the N5 stage were used in the bioassay. dsRNA-treated N5 males were blood-fed (*N* = 20 *dsOBP27*; *N* = 20 *ds*β-*gal*) 7 days after injection. Males were then blood-fed 7 days after molt. Bioassays were conducted 1 week after blood meals. A polystyrene tube (falcon tube) with approximately 10 cm long and 2 cm in diameter was used (**Supplementary Figure S5**). This tube was divided into three zones: female zone (FZ), intermediate zone (IZ), and male release zone (MZ). A gate divides the MZ from IZ. A protective mesh was used to separate MZ and IZ from FZ. An adult female was placed in front of the protection mesh attached by a tape on the tube. Then a male was placed in the MZ and the gate was opened after 5 min of acclimation. The time spent by males to move across the tube toward the female was measured using a digital chronometer and estimated in a maximum period of 300 s. When the insect reached the FZ, the chronometer was reset and started again to record the interval of time that male stayed near the female. The bioassay was repeated 3 times for each insect in each group (*dsOBP27* and *ds*β-*gal*).

### Docking Studies

Since 3D structures have not yet been characterized for *Rhodnius* OBPs, the primary sequence of mature RproOBP27 was used to construct a 3D model for *in silico* docking studies. Three-dimensional modeling was developed using the online protein threading program PHYRE2 (Kelley et al., 2015). Stereochemical quality and accuracy of the predicted model were evaluated using the software PROCHECK (Laskowski et al., 1996) and Verify3D (Eisenberg et al., 1997). The most abundant compounds identified as volatiles emitted by MGs of females and reported as being able to modulate male orientation (Pontes et al., 2008, 2014) were selected for docking studies: 2-methyl-3-buten-2-ol, (2S)-pentanol, (3E)-2-methyl-3-penten-2-ol, and (2R/2S)-4-methyl-3-penten-2-ol. We used thermodynamic principle that ligands tightly bind the active site of a protein when the free binding energy of the process is low (Du et al., 2016). Therefore, such parameter was used to estimate binding affinities of the MGs ligands to RproOBP27. Three-dimensional structures of compounds were obtained from PubChem^2^ (Kim et al., 2016). Molecular docking with RproOBP27 and each of the selected ligands was carried out 100 times using Docking Server (Bikadi and Hazai, 2009) and the free binding-energy scoring function was considered to estimate binding affinity.

### Statistical Analysis

Statistical analysis of qPCRs and bioassays were performed using *t-test* followed by the Mann–Whitney test (GraphPad PRISM 6.00 software, San Diego, CA, United States). qPCRs analyses were done by using three biological and three technique replicates for each gene. Bioassays were carried out independently in three technique replicates. Bars represent the standard error of three replicate, asterisks indicate statistically significant differences (*P* < 0.05).

## Results

### Spatial Expression of *OBPs*

**FIGURE 1:**
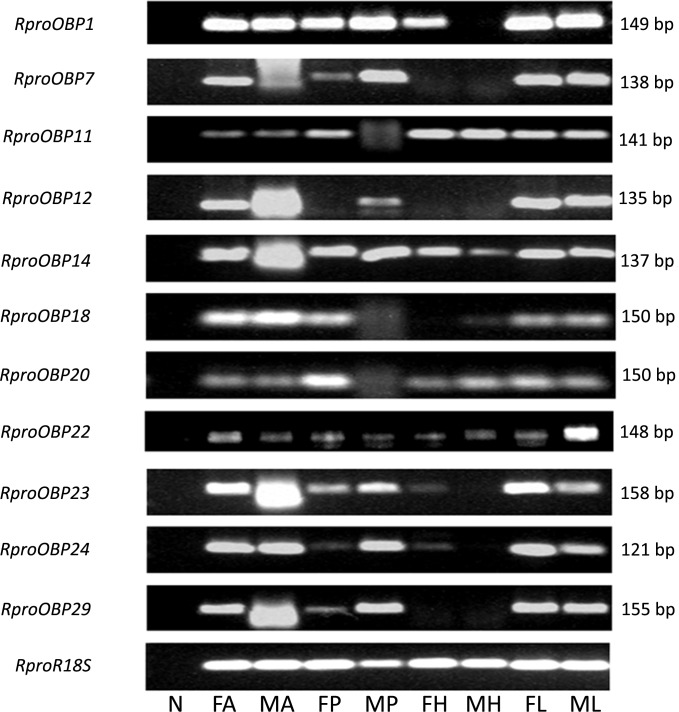
Expression profile of *RproOBP1, RproOBP7, RproOBP11, RproOBP12, RproOBP14, RproOBP18, RproOBP20, RproOBP22, RproOBP23, RproOBP24*, and *RproOBP29* in different *Rhodnius prolixus* tissues evaluated by conventional PCR. N, negative control; FA, female antennae; MA, male antennae; FP, female proboscis; MP, male proboscis; FH, female head; MH, male head; FL, female legs; ML, male legs. *RproR18S* was used as an endogenous control. The amplicon size (bp) is indicated on the right. Heads were used without antennae and proboscis.

**FIGURE 2:**
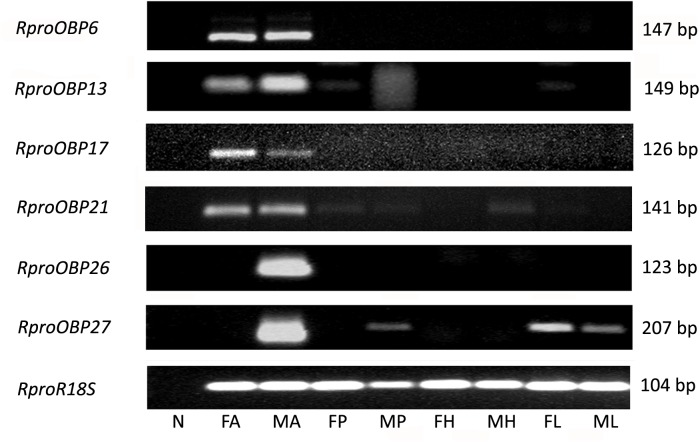
Expression profile of *RproOBP6, RproOBP13, RproOBP17*, *RproOBP21*, RproOBP26, and *RproOBP27* in different *R. prolixus* tissues evaluated by conventional PCR. N, negative control; FA, female antennae; MA, male antennae; FP, female proboscis; MP, male proboscis; FH, female head; MH, male head; FL, female legs; ML, male legs. *RproR18S* was used as an endogenous control. The amplicon size (bp) is indicated on the right. Heads were used without antennae and proboscis.

**FIGURE 3:**
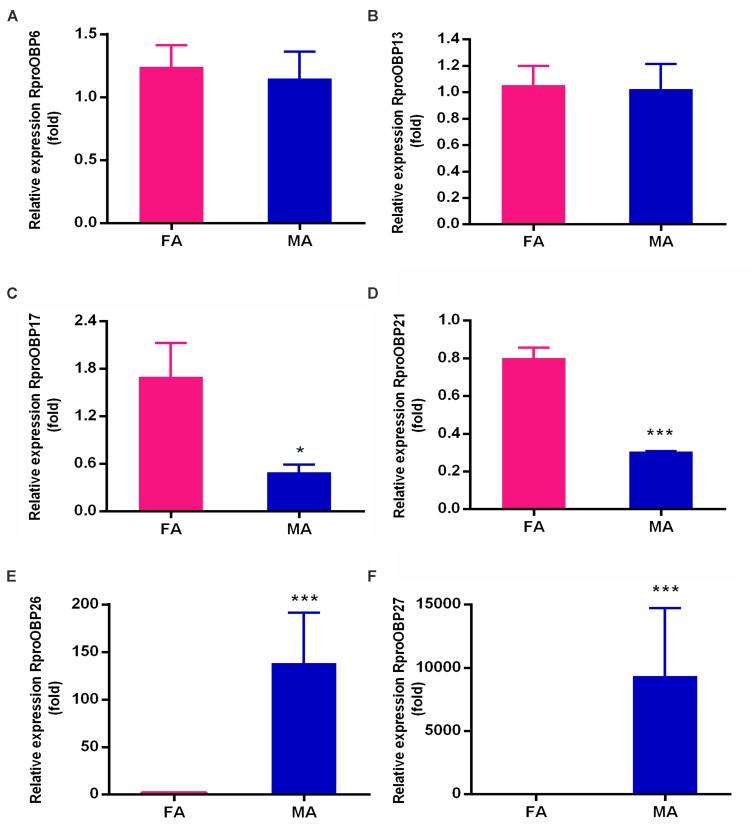
Relative transcript levels of **(A)** *RproOBP6*, **(B)** *RproOBP13*, **(C)** *RproOBP17*, **(D)** *RproOBP21*, **(E)** *RproOBP26*, and **(F)** *RproOBP27* genes in female and male *R. prolixus* antennae, determined by qPCR. The relative expression levels of gene transcripts were compared to the female antennae. Error bars represent standard deviation (SD) of the means of three biological replicates. Statistical analysis was performed using *t-test* followed by the Mann–Whitney test. *RproR18S* was used as an endogenous control. Asterisks represent a significant difference between males and females (*P* < 0.05). FA, female antennae; MA, male antennae.

Previous results showed that 17 OBPs were expressed in the adult antennae (Oliveira et al., 2017), which suggests that at least 17 genes predicted as OBPs in the genome actually encodes antennal functional proteins. In order to investigate which of those 17 OBP genes were antennae-specific, different tissues of adult insects [antennae, proboscis, legs, and heads (without antennae and proboscis)] were screened by PCR. Spatial expression showed that 11 *OBPs* (*RproOBP1, RproOBP7, RproOBP11, RproOBP12, RproOBP14, RproOBP18, RproOBP20, RproOBP22, RproOBP23, RproOBP24*, and *RproOBP29*) were expressed in multiple tissues (**Figure 1**; all original gels appear in **Supplementary Figures**), which strongly suggests that proteins produced by these genes are not specifically related to odorant transport. In contrast, four *OBPs* (*RproOBP6, RproOBP13, RproOBP17*, and *RproOBP21*) were detected specifically in adult antennae, although minor bands for *RproOBP13* and *RproOBP21* were detected in other tissues (**Figure 2**). Two OBPs were highly expressed in the male antennae, *RproOBP26* and *RproOBP27* (**Figure 2**), with minor *RproOBP27* bands being observed in male proboscis and male and female legs (**Figure 2**; see also the original gels in the **Supplementary Figures S1**–**S4**). To further investigate these qualitative profiles, *OBPs* that were found to be enriched in the antenna were quantified by qPCR. Proboscis, legs, and heads (without antenna and proboscis) were also analyzed by qPCR. Considering that we did not identify any transcripts in proboscis, heads, and legs, the above described bands in these tissues (detected by conventional PCR) were probably not specific bands for the tested genes (**Figure 2**). Quantitative results confirmed that *RproOBP6* and *RproOBP13* were expressed exclusively in male and female adult antennae and did not exhibit transcripts in other tissues (**Figures 3A,B**). In addition, *RproOBP17* and *RproOBP21* were enriched in female antennae (*P* < 0.05) (**Figures 3C,D**). On the other hand, *RproOBP26* and *RproOBP27* were shown to have high and specific expression in male antenna (*P* < 0.05) (**Figures 3E,F**).

### Role of *RproOBP27* on Male Behavior

#### Silencing of *RproOBP27*

**FIGURE 4:**
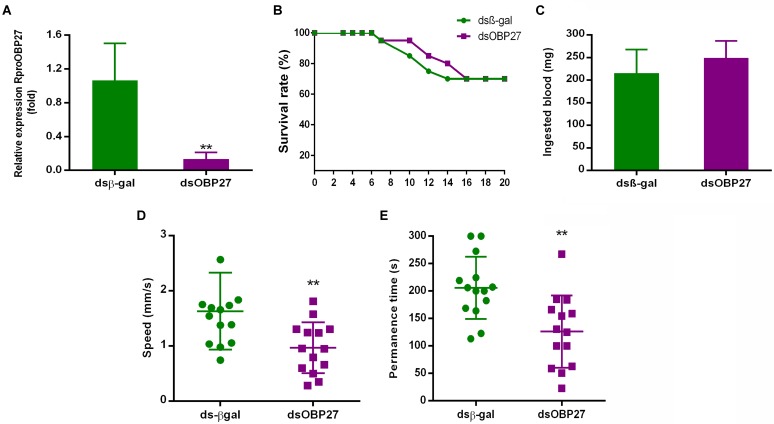
**(A)** qPCR analysis of *RproOBP27* gene in antennae of dsRNA treated adult male. Insects were injected with control *ds*β-*gal* (not related gene) or with *dsOBP27*. The relative expression levels of gene transcripts were compared to the *ds*β-*gal*. *RproR18S* was used as an endogenous control. **(B)** Survival rate of starved N5 males treated with dsRNA (*N* = 20 for *dsOBP27* and *N* = 20 for *ds*β-*gal*). The survival of the insects was monitored from the 3^rd^ to the 20^th^ day after dsRNA injection. **(C)** Blood feeding of insects injected with *dsOBP27* and *ds*β-*gal*. The treated-*dsOBP27* and *ds*β-*gal* N5s were weighed 2 h before and 2 h after blood intake. The ingested mass (mg) was calculated by the weight difference after and before the feed. **(D)** Speed of dsRNA treated male to access female. After molt, the adults injected with *dsOBP27* and *ds*β-*gal* were blood-fed. After 7 days fed insects were individually tested using a polystyrene tube. The time spent by males to arrive close to a caged female was recorded during for up to 300 s. **(E)** Time spent by dsRNA treated male close to female. After molt, the adult injected with *dsOBP27* and *ds*β-*gal* was blood-fed. After 7 days fed insects were individually tested using a polystyrene tube. Time spent by males close to female was recorded for up to 300 s. Error bars represent standard deviation of the means of three biological and technical replicate. Statistical analysis was performed using *t-test* followed by the Mann–Whitney test. Asterisks represent a significant difference (*P* < 0.05).

Next, we reduced the expression of *RproOBP27* using RNAi and evaluated the behavior of the male phenotype. Transcript levels of *RproOBP27* were compared to control *ds*β-*gal*. *RproR18S* was utilized as a reference gene to calculate relative expression. *dsOBP27* injected-group exhibited a significant reduction in *RproOBP27* expression (8x) when compared to control groups (**Figure 4A**). In fact, the *ds*β-*gal* fold change mean was 1.06, while *dsOBP27* was 0.13, which indicates an 88% expression decrease.

#### Effects of Reduction in the Expression of *RproOBP27* on Male Physiology

Insect survival was monitored from 3 to 20 days before molting. The survival index ranged from 70 to 95%, which showed that the injection of *ds*β-*gal* and *dsOBP27* did not affect the insect’s lifespan (**Figure 4B**). Another important aspect of male physiology, which was not affected by dsRNA treatment, was blood feeding. The reduction of *RproOBP27* expression did not affect the ability of male adults to take a blood meal (**Figure 4C**). There was no significant difference (*P* = 0.4206) in blood intake by *dsOBP27*-treated (249.1 ± 16.85 mg/blood, *N* = 5) and *ds*β-*gal*-treated (215.8 ± 23.14 mg/blood, *N* = 5) insects.

#### Behavioral Response of *dsOBP27*-Treated Male

The time spent by the male to move across the tube and reach next to the female was recorded for a period of 300 s. *dsOBP27*-treated insects accessed the FZ (female zone) with a speed of 0.96 ± 0.12 mm/s, whereas *ds*β-*gal*-treated insects responded significantly faster (1.63 ± 0.19 mm/s, *N* = 14, *P* = 0.0065) (**Figure 4D**). *dsOBP27* insect-groups stayed close to females for a significantly (*P* = 0.002) shorter period of time (126.1 ± 17.6 s) than *ds*β-*gal*-treated insects (205.9 ± 15.2 s) (**Figure 4E**). Additionally, we observed that, as opposed to treated insects, control males attempted to copulate with females through the mesh separating them in the arena.

#### 3D Model Prediction and *in silico* Forecasting of RproOBP27 Function

**FIGURE 5:**
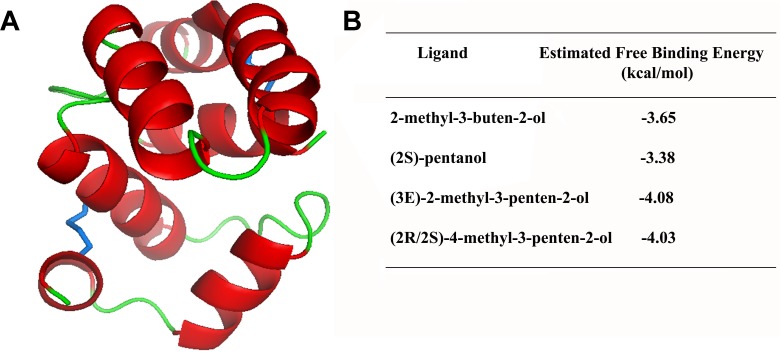
3D model of RproOBP27. α-Helices are shown in red, loops in green and disulfide linkages are highlighted in blue. 3D modeling was developed using the crystal structure of OBP20 from *Anopheles gambiae* (PDB Code: 3VB1) as template by the online program PHYRE2 and validated using PROCHECK and Verify3D.

Using Phyre2, 12 3D models were obtained, including *Antheraea polyphemus* PBP1 [PDB#2JPO; confidence (C) = 99.4; % i.d. = 18]; *Amylois transitella* PBP1 (PDB#4INW; C = 99.4; % i.d. = 18); *Bombyx mori* PBP1 (PDB#1DQE; C = 99.3; % i.d. = 15), OBP2 (PDB#2WCL; C = 99.3; % i.d. = 22), *Leucophaea maderae* PBP (PDB#1OW4; C = 99.1; % i.d. = 10); *Apis melifera* OBP5 (PDB#3R72; C = 99.1; % i.d. = 12); *An. gambiae* OBP4 (PDB#3Q8I; C = 99.0; % i.d. = 17); *A. melifera* OBP14 (PDB#3S0B; C = 98.9; % i.d. = 10), OBP (PDB#1R5R; C = 98.9; % i.d. = 14), *Phormia regina* OBP56a (PDB#5DIC; C = 98.9; % i.d. = 14); *An. gambiae* OBP20 (PDB#3BV1; C = 98.8; % i.d. = 16), and *Locusta migratoria* OBP1 (PDB#4PT1; C = 98.8; % i.d. = 16). Then, PROCHECK and Verify 3D were used to find a model for RproOBP27. The best model for RproOBP27 (**Figure 5A**) was obtained using the crystal structure of OBP20 from *An. gambiae* (AgamOBP20, PDB#3VB1) as template and used in docking studies. This model was the one which best satisfied the criteria required by PROCHECK and Verify 3D in order to validate as a good model (**Supplementary Figures S6**, **S7**). Binding affinities of RproOBP27 were tested against 2-methyl-3-buten-2-ol, (2S)-pentanol, (3E)-2-methyl-3-penten-2-ol and (2R/2S)-4-methyl-3-penten-2-ol. Thermodynamically, ligands tightly bind the active site of a protein when the free binding energy of the process is low. Therefore, such parameter was used to estimate binding affinities of 2-methyl-3-buten-2-ol, (2S)-pentanol, (3E)-2-methyl-3-penten-2-ol and (2R/2S)-4-methyl-3-penten-2-ol to RproOBP27. Negative values suggested favorable interactions with all tested ligands (**Figure 5B**). However, since using a cut-off value of -4.00 results still indicate that RproOBP27 is able to bind (3E)-2-methyl-3-penten-2-ol and (2R/2S)-4-methyl-3-penten-2-ol, both compounds already described as being involved in flight orientation modulated by female-emitted volatiles, a male-specific behavior. The 3D model of RproOBP27 docked with MG volatiles (3E)-2-methyl-3-penten-2-ol and (2R/2S)-4-methyl-3-penten-2-ol appears in **Supplementary Figure S8**. The amino acid sequence of RproOBP27 is displayed in **Supplementary Figure S9**.

## Discussion

Chemical communication is one of the oldest forms of communication used from worms to mammals (Wyatt, 2014; Tomberlin et al., 2016). Insects have a refined olfactory system for the detection of chemical signals from the environment. Chemical signals evoke specific behaviors which allows insects to obtain food, find mates and shelter, and run away from predators. The first contact between the external environment and the internal olfactory machinery occurs when odorants penetrate through the sensillum pores in antennae and reach soluble OBPs found in the sensillar lymph (Brito et al., 2016). Subsequently ORs, IRs, and odorant degrading enzymes are involved. The processing of these semiochemicals ultimately leads to a behavioral response which is essential for insect survival (Leal, 2012). Thus, blocking the first step of the process could be a key factor for controlling insect populations. Research regarding olfactory mechanisms of *R. prolixus* for such purpose only became possible after genome release, when many genes related to chemosensory detection were identified (Mesquita et al., 2015). Here, we present a compilation of data that strongly suggests the role of RproOBP27 in *R. prolixus* behavior.

### Profile of Odorant Binding Protein Genes

Although the genome predicts 27 genes which encode *OBPs* (Mesquita et al., 2015), only 17 OBPs were found to be expressed in adult antennae (Oliveira et al., 2017), which suggests that several genes belonging to the OBP family may not be directly involved in odor transport, as observed in other insects (Pelosi et al., 2018). Moreover, amongst the 11 *OBP* transcripts identified in antenna, leg, proboscis, and head from adults (**Figure 1**), four had already been described in the midgut transcriptome: *RproOBP1*, *RproOBP11*, *RproOBP14*, and *RproOBP24* (Ribeiro et al., 2014). Such evidence favors the assumption that these proteins might be involved in transporting general molecules, not necessarily related to odorant reception. In fact, RproOBP11, known as *Rhodnius* heme-binding protein (RHBP), is responsible for the transport of heme radicals generated from blood digestion, shielding cells from oxidative stress (Dansa-Petretski et al., 1995). Some OBPs, for instance, are important in nutrition as lipids solubilizers and other components of the insect diet (Sanchez-Gracia et al., 2009). Therefore, it was not entirely surprising to find transcripts for *OBPs* distributed in non-olfactory tissues.

Using conventional PCR, six *OBPs* transcripts were specifically expressed in antennae: *RproOBP6*, *RproOBP13*, *RproOBP17*, *RproOBP21*, *RproOBP26*, and *RproOBP27* (**Figure 2**), which suggests these OBPs may, in fact, be associated with odorant transport as it has been reported for other insects (Leal, 2012; Schultze et al., 2012; Sun et al., 2014). Of note, no clear differences were observed in transcript levels of antenna specific OBPs between male and female using conventional PCR (**Figure 2**).

Given that qPCR data showed *RproOBP6* and *RproOBP13* were expressed in male and female antennae (**Figures 3A,B**), it is conceivable that these OBPs are involved in the detection of odorants eliciting common adult behaviors (e.g., host finding). *R. prolixus* belongs to the Reduviidae family, where adults are hematophagous (Guerenstein and Lazzari, 2009; Sant’Anna et al., 2017), therefore, adults need to accurately detect host specific volatiles to acquire their blood meal (Otalora-Luna et al., 2004). Thus, we propose that *RproOBP6* and *RproOBP13* might transport host emanations.

Transcripts for *RproOBP17* and *RproOBP21* were enriched in female antennae (**Figures 3C,D**), indicating these proteins might be involved in female-specific behaviors. This hypothesis is supported by the finding that in the mosquito *Culex quinquefasciatus*, another hematophagous insect, some OBPs expressed in the female antenna are specifically related to the detection of oviposition odorants. OBP2 is postulated to carry the oviposition attractant skatole, whereas OBP1 and OBP5 were implicated in the transport of a mosquito oviposition pheromone (MOP), which induced oviposition behavior in females (Pelletier et al., 2010; Yin et al., 2015).

Lastly, transcripts for *RproOBP26* and *RproOBP27* were found to be significantly expressed in the male antenna (**Figures 3E,F**). These results strongly suggest that these proteins could play a role in male-specific behaviors, such as sex pheromone detection. In the mosquito *Aedes aegypti*, OBP10 is enriched in antennae and wings of adult male and it expression pattern has been suggested to correspond to proteins that may play a role on male chemosensory behavior such as pheromone detection (Bohbot and Vogt, 2005). Although *RproOBP26* was highly expressed in antennae (**Figure 3E**), it was also reported to be overexpressed in the midgut of *R. prolixus* (called RP-3726) (Ribeiro et al., 2014). Here we showed that transcripts for *RproOBP26* were significantly more expressed in male than female antennae (**Figure 3E**). However, proteome studies have found soluble RproOBP26 in both male and the female antennae (Oliveira et al., 2017). Thus, we cannot rule out the possibility that RproOBP26 might be involved in the transport of non-sensorial molecules in the gut, as well as semiochemicals in antennae. Although, only one gene for RproOBP26 has been annotated in the genome (Mesquita et al., 2015), we could not exclude the possibility of *RproOBP26* has alternative splicing, as previously observed for other insect species (Forêt and Maleszka, 2006; Hull et al., 2014).

### Role of OBP27 in *R. prolixus* Behavior

Previously, we have demonstrated a direct correlation between an olfactory protein (Orco) and *R. prolixus* behavior by RNAi (Franco et al., 2016). We then surmised that silencing OBPs might lead to behavioral changes in the phenotype. After all, gene silencing has already been successfully applied to investigate functions of OBPs in other insects (Chen et al., 2008; Pelletier et al., 2010; Rebijith et al., 2016). Of the two OBPs specific to male antennae, we selected *RproOBP27* for these studies. We envisioned that this protein might generate a clearer picture than *RproOBP26* given the possible dual role (or multiple roles) played by RproOBP26 in *R. prolixus* physiology.

Adult males treated with dsOBP27 had a reduction of 88% in *RproOBP27* expression (**Figure 4A**), representing a drastic decrease in the amount of protein circulating in antennae. However, this reduction in gene expression did not interfere with survival or blood-intake, since both groups (control- and *dsOBP27*-insects) ingested almost the same amount of blood (**Figures 4B,C**). Differently, a reduction in expression of odorant coreceptor Orco in *R. prolixus* antenna affected directly the ability of insect to take a blood meal (Franco et al., 2016). Thus, we can suggest that *RproOBP27* is not involved in the host-seeking or blood-intake behavior. Next, we tested whether RNAi treatment would affect male ability to detect females. Insects injected with a control gene were able to detected females and run in their direction faster than *dsOBP27*-treated males (**Figure 4D**). Further, *dsOBP27*-males spent almost 40% less time nearby the female when compared to control insects (**Figure 4E**). In addition, while males from control groups tried to stay close to females, *dsOBP27*-treated insects kept running around the tube, indicating they were not able to detect a female. Based on this clear behavioral difference, we hypothesize that RproOBP27 may be involved in the reception of semiochemicals related to mating finding. This hypothesis is further supported by *in silico* analysis.

Volatile compounds emitted by *R. prolixus* female MGs are known to modulate male orientation and to increase copulation attempts (Pontes et al., 2014). Of the 12 compounds identified in MGs, four are considered putative sex pheromones, namely, 2-methyl-3-buten-2-ol, (2S)-pentanol, (3E)-2-methyl-3-penten-2-ol, and (2R/2S)-4-methyl-3-penten-2-ol (Pontes et al., 2008). Docking results (**Figure 5**) indicate favorable interactions with all four tested ligands due to negative values calculated for free binding energy. Even when a more restricted analysis, based on previous studies for predicting behaviorally active compounds (Jayanthi et al., 2014), is used to estimate binding potential, 2-methyl-3-penten-2-ol and (2R/2S)-4-methyl-3-penten-2-ol still meet the criteria for high binding affinity to RproOBP27. These results further support our hypothesis that RproOBP27 is a carrier of female-derived semiochemicals.

In the Lucerne plant bug, *Adelphocoris lineolatus*, expression of OBP1 is 1.91 times higher in male than in female antennae and this protein was shown to exhibit high binding affinity with two putative pheromone components (Gu et al., 2011b). Recent study suggested that OBP expression could be regulated by nutritional state. In *A. lineolatus* starvation significantly increased expression of *AlinOBP13* in male and female antenna (Sun et al., 2014). Likewise, starved *R. prolixus* males did not express *RproOBP27* (**Supplementary Figure S4B**), which was found only in the antennae of fed males. This dataset is consistent with the findings that unfed males from this species do not respond to sexual signals (Baldwin et al., 1971). Taking together, the evidence presented here strongly suggests that RporoOBP27 is likely involved in the reception of sex pheromone(s).

## Author Contributions

AM and WL designed the project and experiments. DO, NB, and TF performed the experiments. DO, NB, TF, MM, WL, and AM analyzed the data. DO, NB, WL, and AM wrote the paper. DO, NB, TF, MM, WL, and AM revised the paper.

## Conflict of Interest Statement

The authors declare that the research was conducted in the absence of any commercial or financial relationships that could be construed as a potential conflict of interest.

## Footnotes

1 https://www.vectorbase.org/organisms/rhodnius-prolixushttp

2 https://pubchem.ncbi.nlm.nih.gov

## Supplementary Material

FIGURE S1 Expression profile of **(A)** *RproOBP1* and *RproOBP24*; **(B)** *RproOBP26*; **(C)** *RproOBP11* and *RproOBP13*; **(D)** *RproOBP12* and *RproOBP7* in different *R. prolixus* tissues evaluated by conventional PCR. Original 1% agarose gel stained with GelRed^TM^. M, molecular weight; N, negative control; FA, female antennae; MA, male antennae; FP, female proboscis; MP, male proboscis; FH, female head; MH, male head; FL, female legs; ML, male legs.

FIGURE S2 Expression profile of **(A)** *RproOBP14* and *RproOBP6*; **(B)** *RproOBP17*; **(C)** *RproOBP18* and *RproOBP20*; **(D)** *RproOBP21* in different *R. prolixus* tissues evaluated by conventional PCR. Original 1% agarose gel stained with GelRed^TM^. M, molecular weight; N, negative control; FA, female antennae; MA, male antennae; FP, female proboscis; MP, male proboscis; FH, female head; MH, male head; FL, female legs; ML, male legs.

FIGURE S3 Expression profile of **(A)** *RproOBP22*; **(B)** *RproOBP22* and *RproOBP23*; **(C)** *RproOBP22* and *RproOBP26*; **(D)** *RproOBP29* and *RproOBP27* in different *R. prolixus* tissues evaluated by conventional PCR. Original 1% agarose gel stained with GelRed^TM^. M, molecular weight; N, negative control; FA, female antennae; MA, male antennae; FP, female proboscis; MP, male proboscis; FH, female head; MH, male head; FL, female legs; ML, male legs.

FIGURE S4 Expression profile of **(A)** *RproR18S*; **(B)** *RproOBP27, RproOBP26*, and *RproOBP21* in different *R. prolixus* tissues evaluated by conventional PCR. Original 1% agarose gel stained with GelRed^TM^. M, molecular weight; N, negative control; FA, female antennae; MA, male antennae; FP, female proboscis; MP, male proboscis; FH, female head; MH, male head; FL, female legs; ML, male legs.

FIGURE S5 Device used in female recognition bioassay. A polystyrene tube (10 × 2 cm) divided into three zones: female zone (FZ), intermediate zone (IZ), and male release zone (MZ). A gate divides the MZ from IZ. A protective mesh was used to separate MZ and IZ from FZ. An adult female was placed in front of the protection mesh attached by a tape on the tube. Then a male was placed in the MZ and the gate was opened. Adapted from Zermoglio et al. (2015).

FIGURE S6 PROCHECK results from predicted 3D model of RproOBP27.

FIGURE S7 Verify3D results from predicted 3D model of RproOBP27.

FIGURE S8 3D model of RproOBP27 docked with metasternal gland volatile compounds (putative sex pheromones). **(A)** RproOBP27 docked with (3E)-2-methyl-3-penten-2-ol. **(B)** RproOBP27 docked with (2R/2S)-4-methyl-3-penten-2-ol.

FIGURE S9 RproOBP27 sequence. The signal peptide is highlighted in red.

TABLE S1 Oligonucleotides used in the PCR, qPCR and dsRNA synthesis reactions.
