# Supplementary figures and images for: Functional Characterization of Odorant Binding Protein 27 (RproOBP27) From Rhodnius prolixus Antennae
Source: Front Physiol. 2018 Aug 23;9:1175. doi: 10.3389/fphys.2018.01175 (PMC6119777; doi:10.3389/fphys.2018.01175)

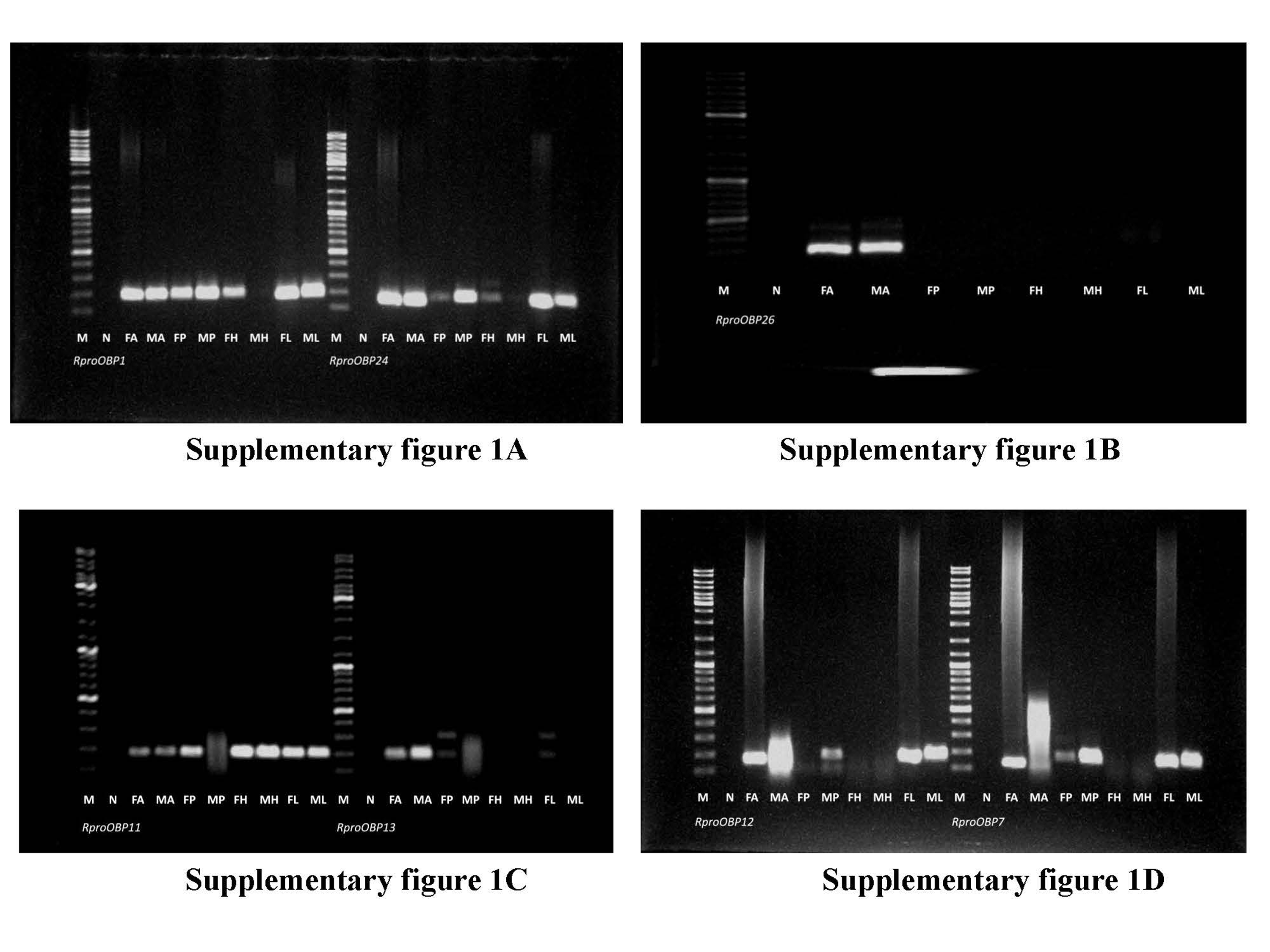

Supplement: FIGURE S1 — Expression profile of (A) RproOBP1 and RproOBP24; (B) RproOBP26; (C) RproOBP11 and RproOBP13; (D) RproOBP12 and RproOBP7 in different R. prolixus tissues evaluated by conventional PCR. Original 1% agarose gel stained with GelRedTM. M, molecular weight; N, negative control; FA, female antennae; MA, male antennae; FP, female proboscis; MP, male proboscis; FH, female head; MH, male head; FL, female legs; ML, male legs. [file Image_1.tif]

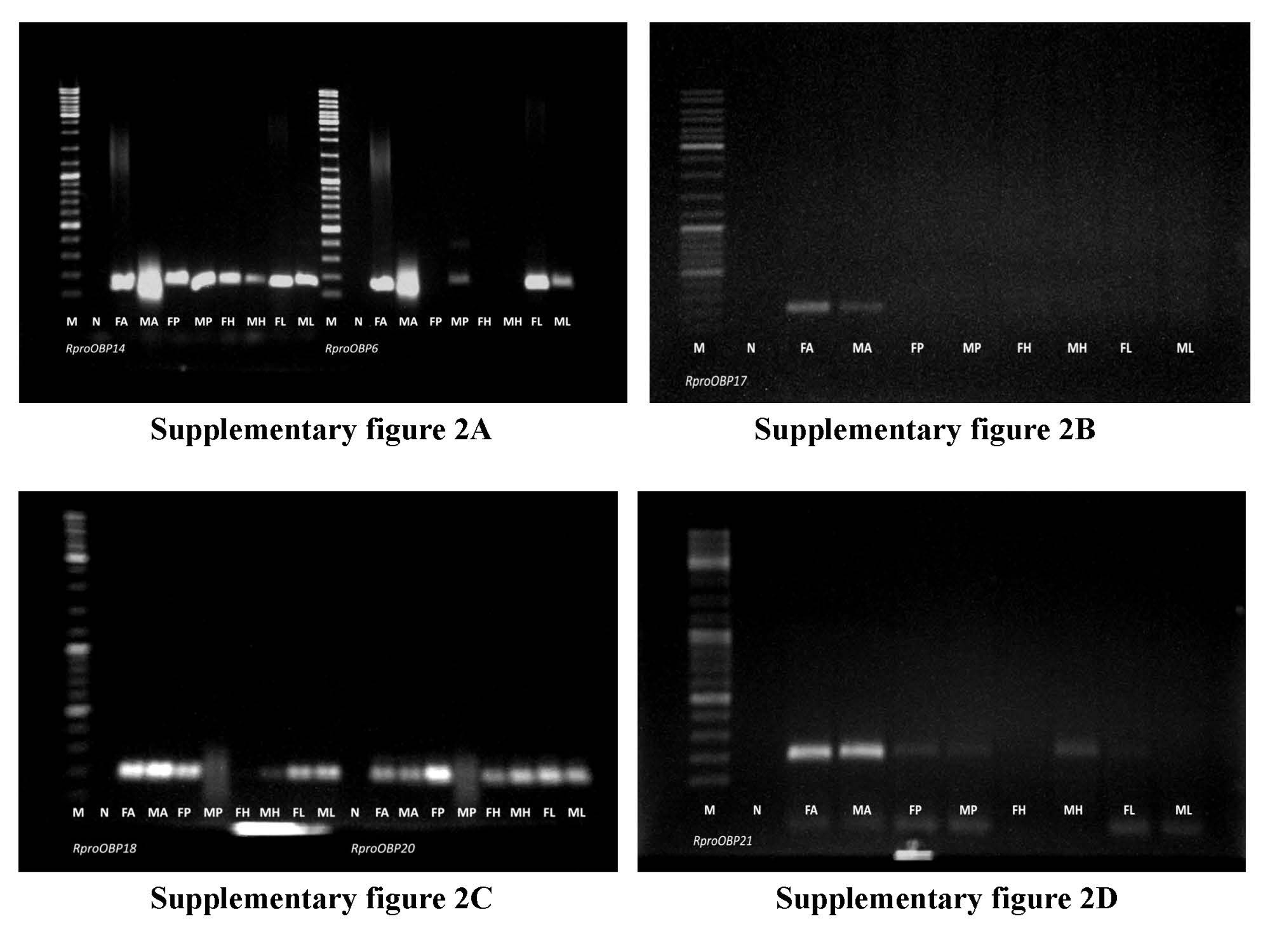

Supplement: FIGURE S2 — Expression profile of (A) RproOBP14 and RproOBP6; (B) RproOBP17; (C) RproOBP18 and RproOBP20; (D) RproOBP21 in different R. prolixus tissues evaluated by conventional PCR. Original 1% agarose gel stained with GelRedTM. M, molecular weight; N, negative control; FA, female antennae; MA, male antennae; FP, female proboscis; MP, male proboscis; FH, female head; MH, male head; FL, female legs; ML, male legs. [file Image_2.tif]

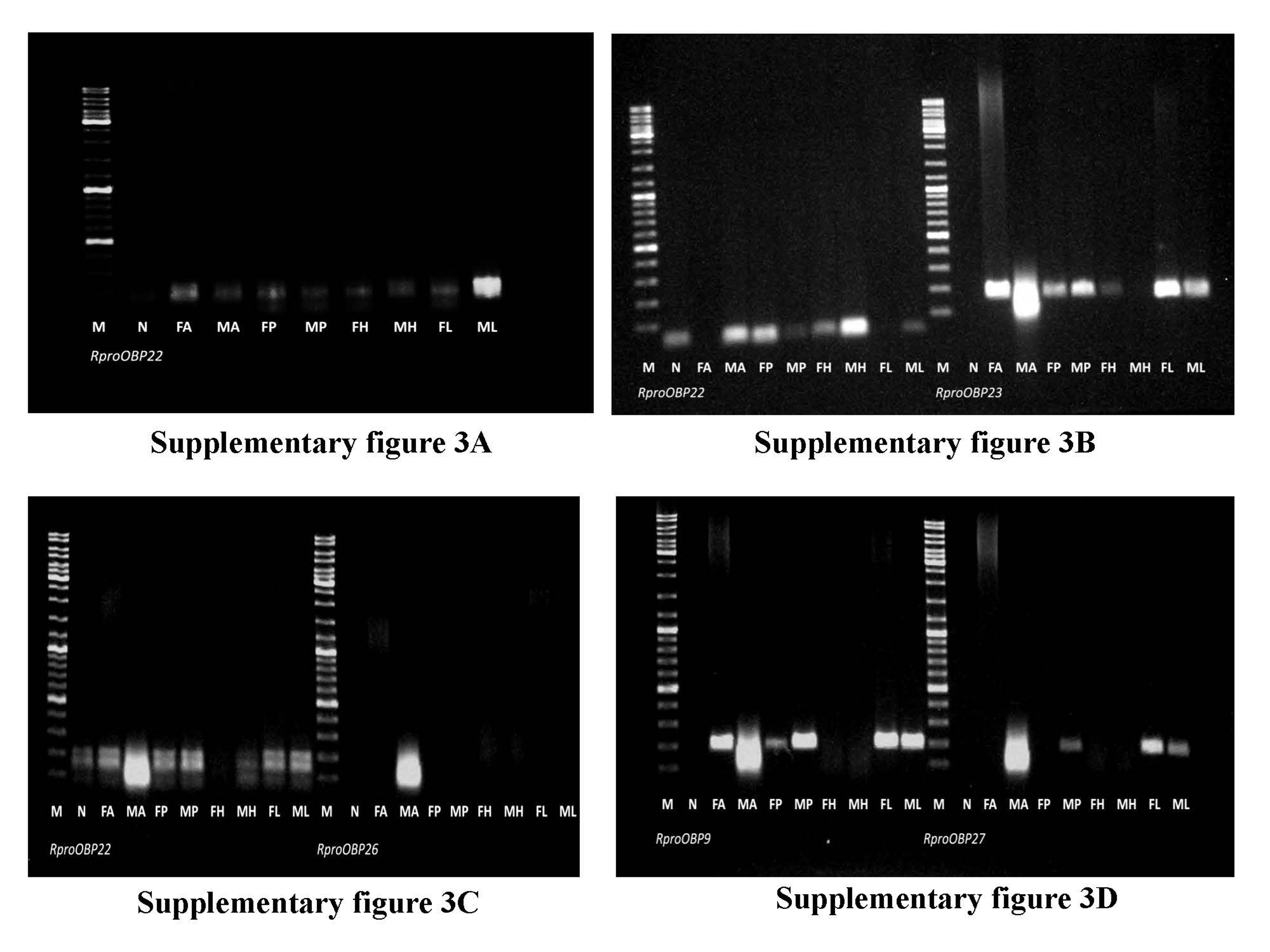

Supplement: FIGURE S3 — Expression profile of (A) RproOBP22; (B) RproOBP22 and RproOBP23; (C) RproOBP22 and RproOBP26; (D) RproOBP29 and RproOBP27 in different R. prolixus tissues evaluated by conventional PCR. Original 1% agarose gel stained with GelRedTM. M, molecular weight; N, negative control; FA, female antennae; MA, male antennae; FP, female proboscis; MP, male proboscis; FH, female head; MH, male head; FL, female legs; ML, male legs. [file Image_3.tif]

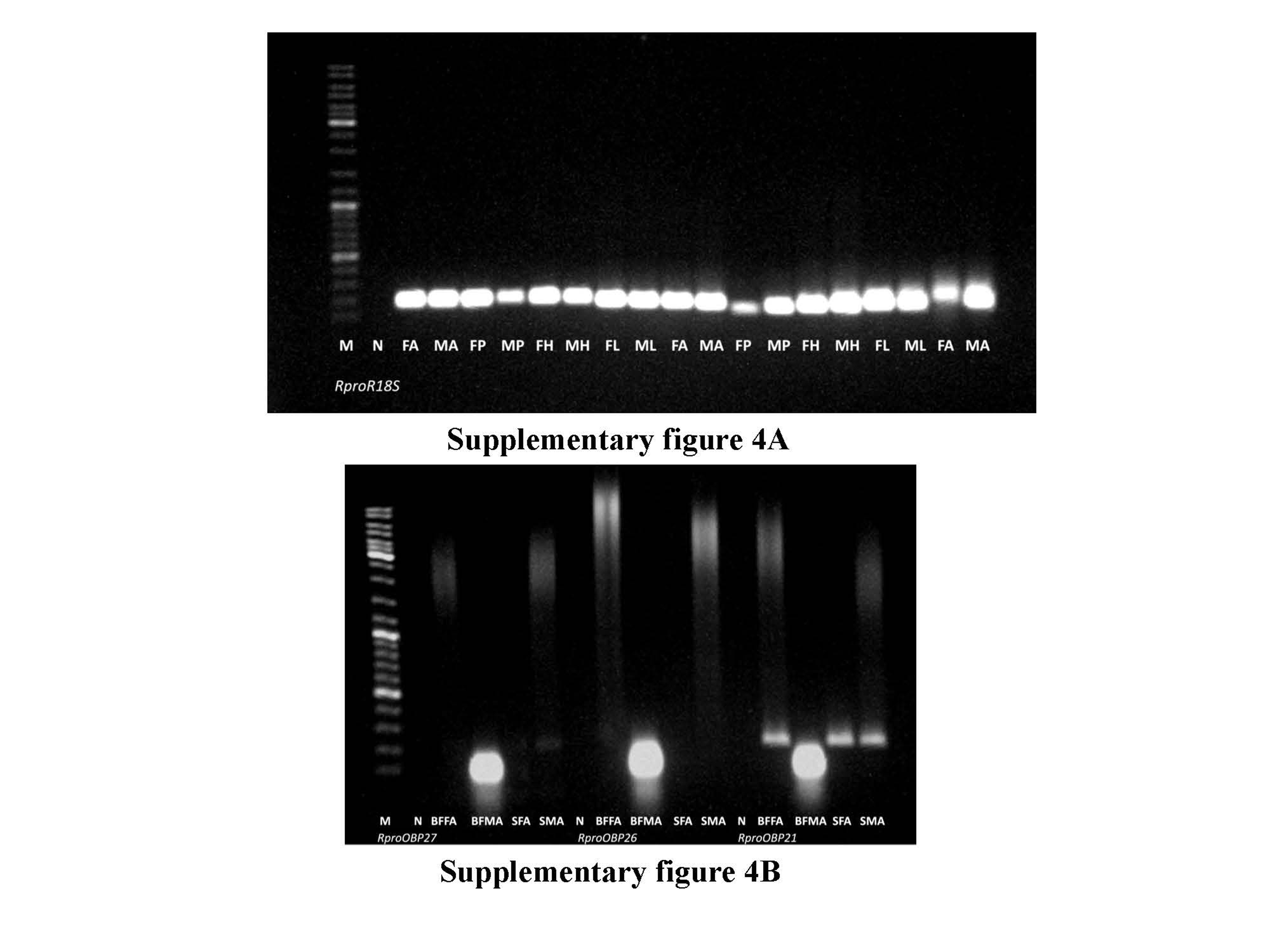

Supplement: FIGURE S4 — Expression profile of (A) RproR18S; (B) RproOBP27, RproOBP26, and RproOBP21 in different R. prolixus tissues evaluated by conventional PCR. Original 1% agarose gel stained with GelRedTM. M, molecular weight; N, negative control; FA, female antennae; MA, male antennae; FP, female proboscis; MP, male proboscis; FH, female head; MH, male head; FL, female legs; ML, male legs. [file Image_4.tif]

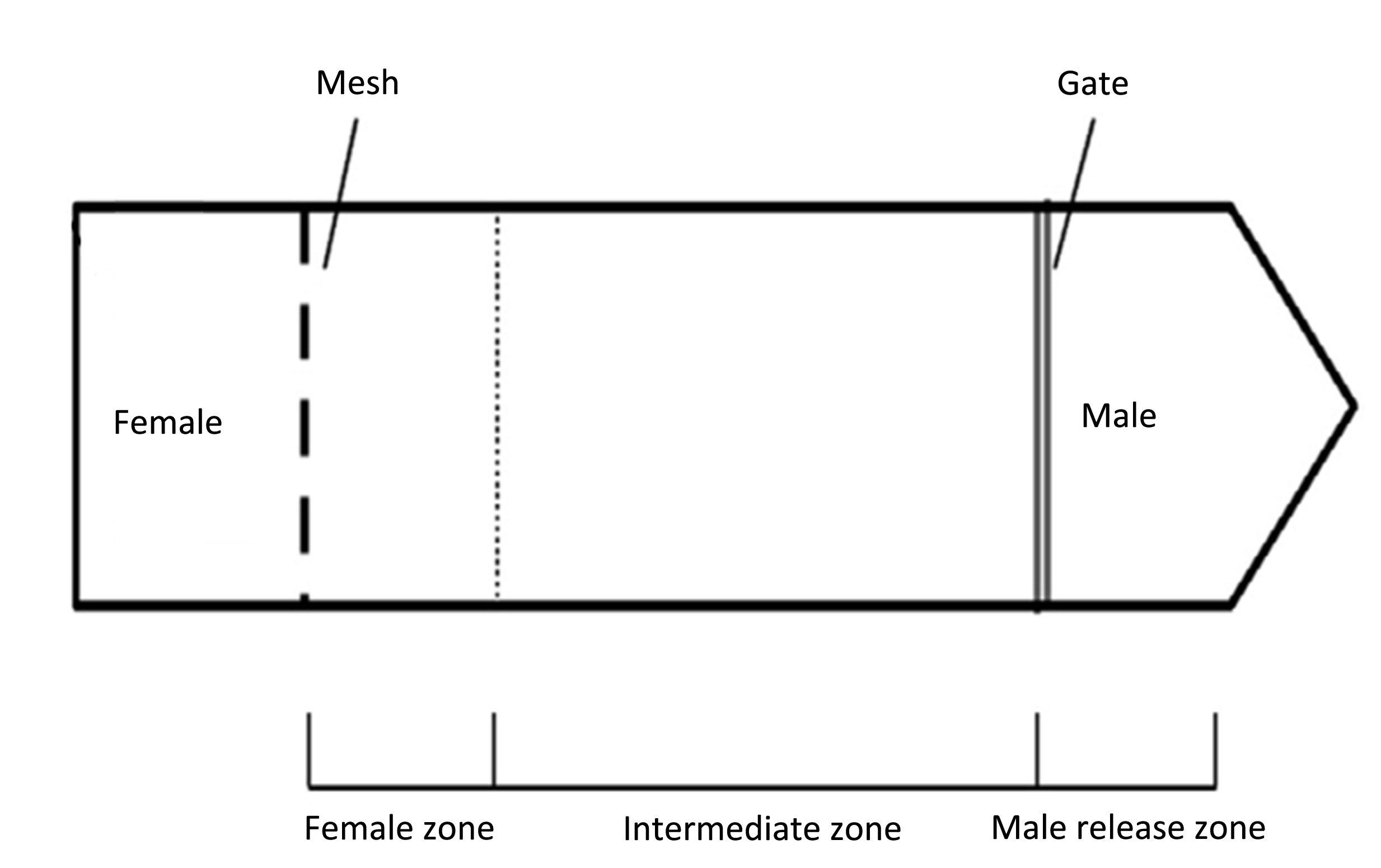

Supplement: FIGURE S5 — Device used in female recognition bioassay. A polystyrene tube (10 × 2 cm) divided into three zones: female zone (FZ), intermediate zone (IZ), and male release zone (MZ). A gate divides the MZ from IZ. A protective mesh was used to separate MZ and IZ from FZ. An adult female was placed in front of the protection mesh attached by a tape on the tube. Then a male was placed in the MZ and the gate was opened. Adapted from Zermoglio et al. (2015). [file Image_5.TIF]

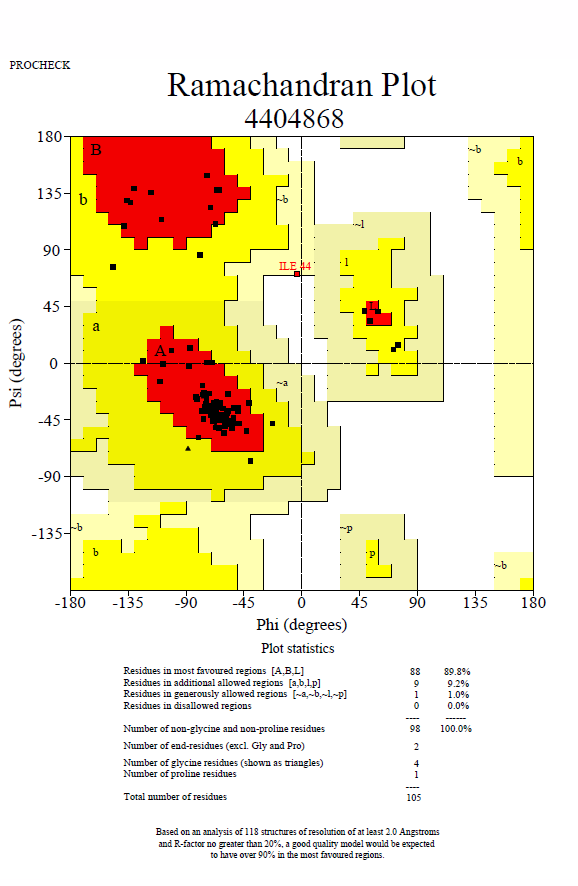

Supplement: FIGURE S6 — PROCHECK results from predicted 3D model of RproOBP27. [file Image_6.TIF]

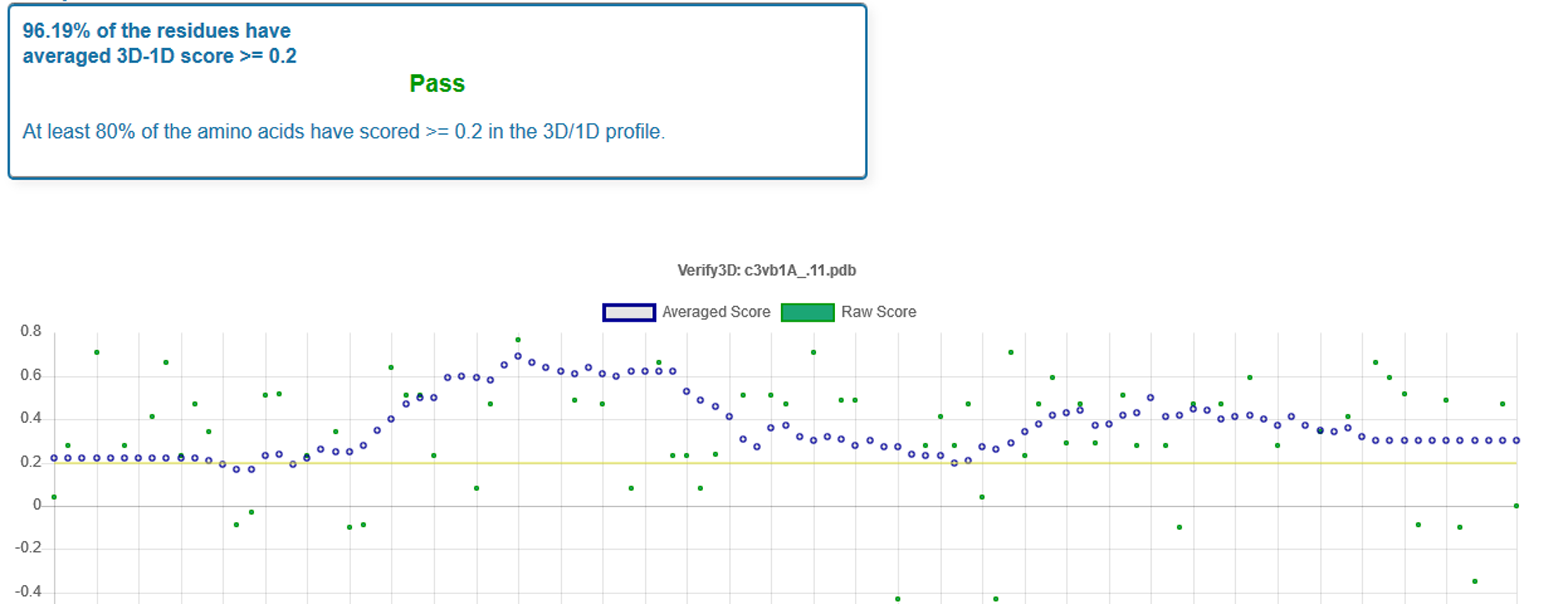

Supplement: FIGURE S7 — Verify3D results from predicted 3D model of RproOBP27. [file Image_7.TIF]

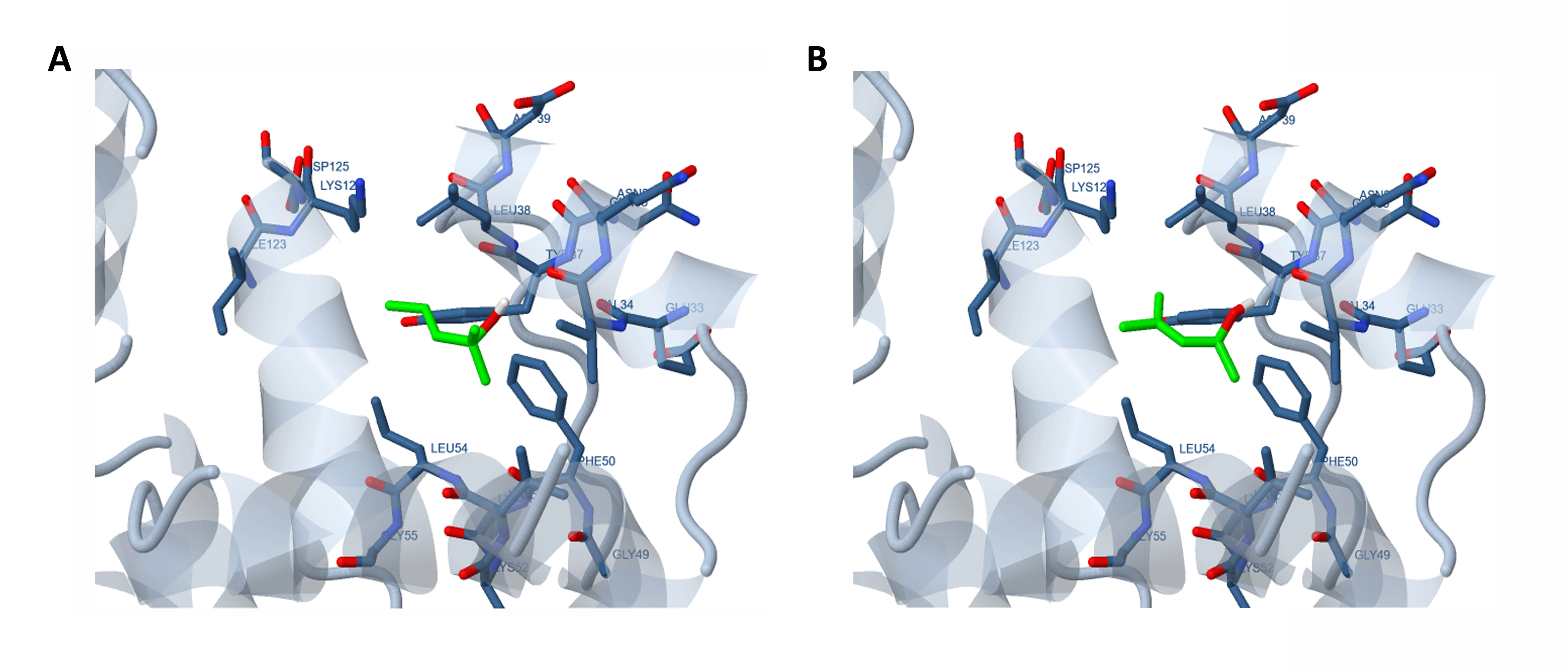

Supplement: FIGURE S8 — 3D model of RproOBP27 docked with metasternal gland volatile compounds (putative sex pheromones). (A) RproOBP27 docked with (3E)-2-methyl-3-penten-2-ol. (B) RproOBP27 docked with (2R/2S)-4-methyl-3-penten-2-ol. [file Image_8.TIF]

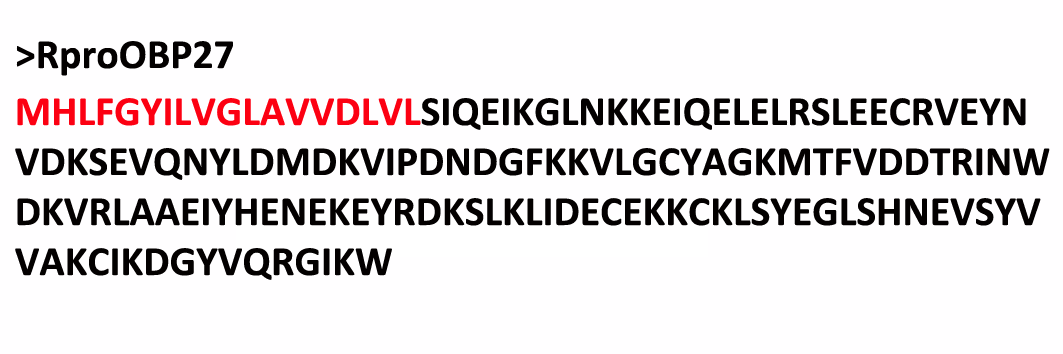

Supplement: FIGURE S9 — RproOBP27 sequence. The signal peptide is highlighted in red. [file Image_9.TIF]
