# Supplementary material for: Functional Characterization of Odorant Binding Protein 27 (RproOBP27) From Rhodnius prolixus Antennae
Source: Front Physiol. 2018 Aug 23;9:1175. doi: 10.3389/fphys.2018.01175 (PMC6119777; doi:10.3389/fphys.2018.01175)
Supplement: TABLE S1 — Oligonucleotides used in the PCR, qPCR and dsRNA synthesis reactions. [file Table_1.docx]

| **Supplementary table 1 -** Primers used in PCR, qPCR and RNAi reactions. | | | | |
| --- | --- | --- | --- | --- |
| **Gene Name / VectorBase ID** | **Primer** | **Sequence** | **Tm ºC** | **Amplicon size (bp)** |
| *RproOBP1* RPRC010284 | Forward | CCGCCACACAAAGCCCTG | 63,1 | 149 |
|  | Reverse | GACACATTCCAGCAAACACCTT |  |  |
| *RproOBP6* RPRC004413 | Forward | GCACAAATGAAACAGGCGATGAA | 63,1 | 147 |
|  | Reverse | GCCCATAATACAGCCTAAGTAGCA |  |  |
| *RproOBP7* RPRC000311 | Forward | AAAGAAATGCCAACAACTTTCCA | 60,8 | 138 |
|  | Reverse | GCTGCTCCAATCATCAATCCA |  |  |
| *RproOBP11* RPRC004408 | Forward | GCAGCACTAAGCACCATTTATC | 60,0 | 141 |
|  | Reverse | AATCTACTCTCCCATCAACGAG |  |  |
| *RproOBP12* RPRC000492 | Forward | ATGGCAAACCATGTGTAGAGG | 60,9 | 135 |
|  | Reverse | CCAATTCTCCTTCTTCTTTAGCTTTC |  |  |
| *RproOBP13* RPRC000177 | Forward | GGAGTGCCATAGAGGAAGTG | 59,8 | 149 |
|  | Reverse | GTAACAAGAATATAGAGCGAACCC |  |  |
| *RproOBP14* RPRC004407 | Forward | CTAAACTAGCAGAGCTTGAGGCA | 62,8 | 137 |
|  | Reverse | GCAGCCGAGTACACACTTGA |  |  |
| *RproOBP17* RPRC000118 | Forward | TGGAGCTTTGATTGAACCTTGT | 60,7 | 126 |
|  | Reverse | GCTTCAGCATCTAATTCACCATC |  |  |
| *RproOBP18* RPRC001924 | Forward | CAGTGCTGACATGGCTTCTA | 59,8 | 150 |
|  | Reverse | GGTCTGGTAACTCCGATCTTT |  |  |
| *RproOBP20* RPRC007008 | Forward | ATAGCGACAACGCCGAAG | 59,9 | 150 |
|  | Reverse | CCAGTCCAATCTCTTTAGCCT |  |  |
| *RproOBP21* RPRC001925 | Forward | TCCAGAAACTGTGCAATACCTT | 60,6 | 141 |
|  | Reverse | TCACCATCATCTGACAACATACC |  |  |
| *RproOBP22* RPRC000115 | Forward | GGCATCCCAAATGAGAGGTT | 60,6 | 148 |
|  | Reverse | CAGGCAACGGTACACTTTCA |  |  |
| *RproOBP23* RPRC000174 | Forward | AAAGACGCCCAGCCACAA | 62,0 | 158 |
|  | Reverse | GCCATAGAATAACCAAGTTCACAGA |  |  |
| *RproOBP24* RPRC009905 | Forward | GCTCTAGCTGTTGCTTCAGACC | 63,6 | 121 |
|  | Reverse | CAAGTTCGCATTCATTATCCATCTTCC |  |  |
| *RproOBP26* RPRC000182 | Forward | TGGGAGAGGTTAAGAGAAAGCTAC | 62,1 | 123 |
|  | Reverse | CTCTTGATAACTTGGATGGAAACGAA |  |  |
| *RproOBP27* RPRC009914 | Forward | TTCAAGAAATTAAAGGGCTTAACAAA | 59,0 | 146 |
|  | Reverse | GCCGTCGTTATCGGGTAT |  |  |
| *RproOBP29* RPRC005254 | Forward | ACTGGGCACGCAATACTC | 59,5 | 155 |
|  | Reverse | CCGCAACCTCTCCATACATA |  |  |
| *RproOBP27*  RNAi | Forward* | TAATACGACTCACTATAGGGAAAGGGCTTAACAAAAAGGAA | 58,2 | 207 |
|  | Reverse* | TAATACGACTCACTATAGGGTTTCCAGCATAACATCCCAAT |  |  |
| *CquiΒ-gal* (XU *et al.,* 2014) | Forward* | TAATACGACTCACTATAGGGAATGGTTCAGGTCGAAAACG | 56 | 500 |
|  | Reverse* | TAATACGACTCACTATAGGGCCGCCTCGTACAAAACAAGT |  |  |
| *RproR18S* (MAJEROWICZ *et al*., 2011) | Forward | TCGGCCAACAAAAGTACACA | 53,4 | 104 |
|  | Reverse | TGTCGGTGTAACTGGCATGT |  |  |
| *The underlined sequences correspond to the T7 promoter sequence. | | | | |
